# Effect of Nitrogen and Phosphorus Fertilizers on Dry Matter Accumulation and Translocation of Two Amylose Content *Indica* Rice on Yield

**DOI:** 10.3390/plants14101536

**Published:** 2025-05-20

**Authors:** Xiaohong Qin, Xinyue Rao, Hongjing Liu, Jiale Hong, Wanlin Tang, Shengmin Yan, Guotao Yang, Hong Chen, Yungao Hu

**Affiliations:** 1 School of Life Science and Agri-Forestry, Southwest University of Science and Technology, Mianyang 621010, China; qinxiaohong20@163.com (X.Q.); raoxinyue2024@163.com (X.R.); lhj20171231lsw@outlook.com (H.L.); 15882499717@163.com (J.H.); tangwanl2025@163.com (W.T.); yangguotao@163.com (G.Y.); 2 Zigong Academy of Agricultural Sciences, Zigong 643002, China; yan_shengmin@126.com

**Keywords:** dry matter accumulation, yield, different rectilinear starch contents, nitrogen and phosphorus utilization rate, stem and leaf dry matter translocation

## Abstract

Nitrogen (N) and phosphorus (P) are key factors affecting rice yield. To study the effects of single application of nitrogen, phosphorus and their combined application on dry matter accumulation and yield of rice, two types of *indica* rice with contenting amylose contents, low amylose content (LAC) and high amylose content (HAC) were used as the test materials. Four different levels of nitrogen and phosphorus were applied (N0: 0, N1: 90, N2: 150, N3: 270 kg/hm^2^) and (P0: 0, P1: 15, P2: 30, P3: 60 kg/hm^2^). The application of N fertilizer alone and in combination with P effectively promote dry matter accumulation, translocation and increase yield. Under the N3P0 and N3P1 treatments, LAC and HAC achieved their highest yield of 10.03 t/hm^2^ and 11.24 t/hm^2^, respectively. representing increased of 46.19% and 29.05% compared to N0P0 treatment. Phosphorus application influenced dry matter accumulation at maturity and stem and leaf dry matter translocation to the panicle, translocation rates, and their contribution to the panicle, there by increasing yield. Effective panicles, spikelets per panicle, grain filling, stem and leaf dry matter translocation, stem and leaf dry matter translocation rate were significantly or highly significantly positively correlated with yield, and 1000-grain weight was highly significantly negatively correlated with yield, which were mainly increased by increasing panicle dry matter accumulation at maturity, the increase in the amount of increase in dry matter of panicle, the contribution rate of stem and leaf dry matter translocation to the panicle, the amount of stem and leaf dry matter translocation, and the rate of stem and leaf dry matter translocation to increase spikelets per panicle and the grain filling, and then to improvement of yield.

## 1. Introduction

Rice (*Oryza sativa* L.) is one of the three major food crops in the world and is the main food crop for more than half of the Chinese population [1]. In order to achieve high and stable yield of rice, the demand for fertilizers has gradually increased [2]. However, there is a certain degree of blindness in the application of fertilizers by farmers [3], and over-application of fertilizers directly or indirectly affects the aggravation of diseases in rice, lower yields and lower fertilizer utilization, resulting in the imbalance of soil nutrient structure and eutrophication of the water body [4,5]. Improving the efficient use of fertilizer is an important issue in the rice production process and an important factor affecting the high yield and quality of rice [6,7].

Nitrogen plays an important role in promoting rice dry matter accumulation and yield enhancement [8], while rice dry matter accumulation and translocation are key processes in determining yield [9,10]. During rice growth, the production and storage of dry matter in different organs are closely related to nitrogen utilization, and dry matter is mainly produced through photosynthesis and accumulated in different parts of leaves, stem-sheath, and panicle, etc. [11]. Proper nitrogen fertilization increases dry matter accumulation at flush, dry matter accumulation from flush to maturity, translocation rate and yield [12]. At rice maturity, nutrients accumulated in the early stages are transported from the nutrient organs to the grains, which is crucial for the formation of grain filling and yield [13,14].

Phosphorus is a constituent of adenosine triphosphate (ATP), which plays an important role in photosynthesis and respiration, and lack of phosphorus fertilizer may inhibit the photosynthetic phosphorylation process and insufficient supply of energy (ATP), leading to late maturity of crops and reduced grain filling [15]. The amount of phosphorus applied not only has a major impact on rice uptake, but also affects the crop’s uptake and utilization of other nutrients, which ultimately affects yield [16]. The uptake of phosphorus in rice is lower than that of nitrogen fertilizer, but there is a higher uptake of phosphorus in rice during the period of gestation to tasseling [17]. Rice requires phosphorus uptake at all periods, and continuous phosphorus supply from the tillering stage to the panicle differentiation stage [18,19]. Excessive application of phosphorus fertilizer is detrimental to the growth and development of rice plants, resulting in stunted plant growth and lower yields [20,21].

There are differences in the best fertilization treatments for different types of rice varieties, and the same optimal fertilizer model cannot meet the needs of all types of rice varieties. Finding the optimal fertilizer pattern according to the demand of different types of rice varieties is important for high yield and quality of rice and reducing pollution in agricultural production [22,23]. There have been more studies on the effects of nitrogen or phosphorus fertilizers individually on dry matter and yield of low or high amylose content rice, respectively [24,25,26]. However, fewer studies have been conducted on the effects of nitrogen and phosphorus individually and nitrogen-phosphorus fertiliser in combination application treatments on dry matter and yield of low and high amylose content rice, respectively. The growth and development of different types of rice varieties were explored under the same treatment. Accordingly, this study was conducted to investigated the effects of four nitrogen and phosphorus levels on the growth and development of two types of *indica* rice with straight chain starch content, to provide some guidance on the use of fertilizers and high yield and quality cultivation of different types of rice varieties.

## 2. Results

### 2.1. Effect of Nitrogen and Phosphorus Fertilizers on Dry Matter Accumulation in Rice

**Figure 1:**
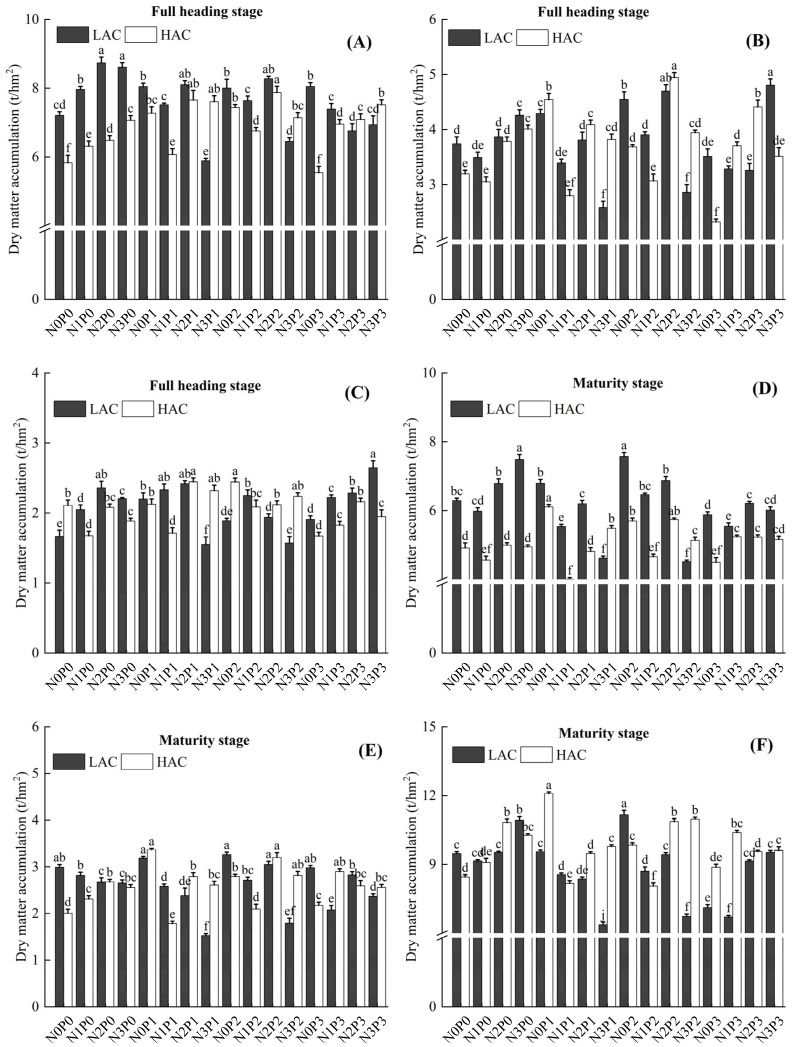
Effect of nitrogen and phosphorus fertilizers on dry matter accumulation in different parts of full heading and maturity. (**A**) stem-sheath dry matter accumulation at full heading stage, (**B**) leaf dry matter accumulation at full heading stage, (**C**) panicle dry matter accumulation at full heading stage, (**D**) stem-sheath dry matter accumulation maturity stage, (**E**) leaf dry matter accumulation at maturity stage, (**F**) panicle dry matter accumulation at maturity stage. ±: the standard deviations for the mean values. LAC: variety with low amylose content; HAC: variety with high amylose content; N means applying different nitrogen fertilizers, N0: 0 kg/hm^2^, N1: 90 kg/hm^2^, N2: 150 kg/hm^2^, N3: 270 kg/hm^2^; P means applying different phosphate fertilizers, P0: 0 kg/hm^2^, P1: 15 kg/hm^2^, P2: 30 kg/hm^2^, P3: 60 kg/hm^2^. Different lowercase letters represent significant differences at the *p* < 0.05 level.

At different N levels, phosphorus application mainly affected total dry matter accumulation (TDMA) at the flush and maturity stages of rice (Figure 1). The effects of different phosphorus application rates on TDMA of low amylose content (LAC) and high amylose content (HAC) showed different trends. The TDMA of LAC at the phosphorus-free (P0) level increased with increasing nitrogen application, whereas the TDMA of HAC at the P0 and medium-phosphorus (P2) levels, and of LAC and HAC at the low-phosphorus (P1) and high-phosphorus (P3) levels decreased with increasing nitrogen application, HAC had the highest TDMA of 35.50 t/hm^2^ at N0P1 level. At P2 level, LAC showed an increasing and then decreasing trend with increasing N application, with the highest TDMA of 36.43 t/hm^2^ at N0P2 level.

### 2.2. Effects of Nitrogen and Phosphorus Fertilizers on Dry Matter Transport in Various Organs of Rice at the Tassel Stage–Maturity Stage

At the same phosphorus level, LAC and HAC showed a trend of increasing and then decreasing stem and leaf dry matter translocation (SLDM), stem and leaf dry matter translocation rate (SLDMR) and contribution rate of stem and leaf dry matter translocation to the panicle (CSLDP) with increasing N application (Table 1). LAC and HAC increased SLDM by 19.50% to 45.21% and 24.31% to 77.01% in N2 treatment under P1 treatment, respectively. Increase in dry matter of panicle (IDMP) in LAC and HAC panicle was significantly higher at N0P2 and N0P1, respectively, and increased by 18.82% and 57.10%, respectively, over the N0P0 treatment. The contribution rate of photosynthesis to the panicle (CPP) in both types of rice at P0, P1 and P2 levels showed a decreasing trend with increasing N application.

### 2.3. Effect of Nitrogen and Phosphorus Fertilizers on Fertilizer Utilization in Rice

LAC and HAC had higher nitrogen harvest index (NHI) under P0 and P2 treatments than P1 and P3 at N0 level (Table 2). Higher nitrogen fertilizer utilization (NAE) and nitrogen fertilizer contribution (NCR) were obtained when P3 was applied at low (N1) and medium (N2) nitrogen levels, which were significantly higher than those of other P treatments, but the difference in terms of nitrogen fertilizer uptake efficiency (NUE) was not significant at P levels. Increased phosphorus fertilization at high nitrogen (N3) levels had no significant effect on NAE and NCR. The results showed that NUE increased with increasing nitrogen application and phosphorus application was not significant on NUE. NAE and NCR were highest at high phosphorus and low nitrogen levels, and phosphorus application had a significant effect on NAE and NCR.

At the P1 and P3 levels, the PHI of the two types of varieties first increased and then decreased with the increase of nitrogen application rate (Table 3). At P1 level, LAC PHI was significantly higher than other N treatments under N3 treatment with a significant increase of 47.12%, and HAC was significantly higher than other N treatments under N1 treatment with an increase of 47.84%; Phosphorus fertilizer utilization (PAE) and phosphorus fertilizer contribution (PCR) showed a tendency to increase and then decrease with increasing N application at the same phosphorus level, and at the P3 level, both PAE and PCR of LAC and PAC reached the highest under the N1 treatment, which was significantly higher than the other N treatments. Phosphorus fertilizer uptake efficiency (PUE) of LAC and HAC increased with increasing N application in all treatments except at the P1 level, where LAC increased with increasing N application at the P3 level, while HAC showed a decreasing trend. The results showed that NUE increased with increasing nitrogen application and phosphorus application was not significant for PUE. PAE and PCR were highest at high phosphorus and low nitrogen levels, and phosphorus application had a significant effect on PAE and PCR.

### 2.4. Effect of Nitrogen and Phosphorus Fertilizers on Rice Yield and Yield Components

LAC and HAC showed the same performance in terms of yield components at different levels of nitrogen and phosphorus (Table 4). The number of effective panicles of LAC and HAC increased with increasing N application at P0 and P1 levels, whereas LAC and HAC first increased and then decreased with increasing N application at P2 and P3 levels. At the same phosphorus level LAC and HAC fruiting percentage and thousand grain weight showed an increasing and then decreasing trend with increasing N application.

Nitrogen phosphorus fertilizers were significantly different on the yield of both types of rice. LAC at the P0 level, yield of both types of rice increased with increasing N application at P1 and P2 levels, among them, LAC had the highest yield of 10.03 t/hm^2^ at N3P0 level and HAC had the highest yield of 11.24 t/hm^2^ at N3P1 level. At P3 level the yields of both types of rice increased firstly and then decreased with the increase of N application.

### 2.5. Effects of Nitrogen and Phosphorus Fertilizers on Enzyme Activities Related to Nitrogen Metabolism in Rice

**Figure 2:**
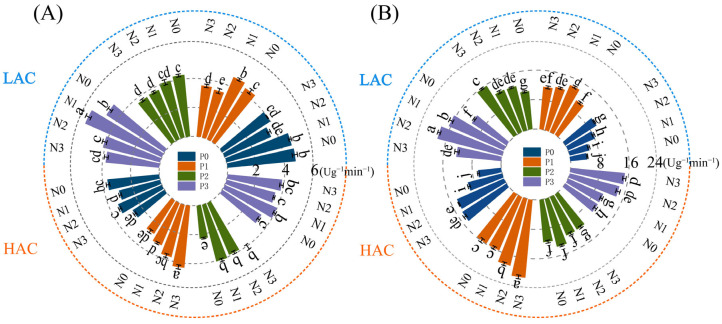
Effect of nitrogen and phosphorus fertilizers on enzyme activities related to nitrogen metabolism in rice. (**A**) Nitrate reductase activity of LAC and HAC; (**B**) Glutamine synthetase of LAC and HAC. ±: the standard deviations for the mean values. LAC: variety with low amylose content; HAC: variety with high amylose content; N means applying different nitrogen fertilizers, N0: 0 kg/hm^2^, N1: 90 kg/hm^2^, N2: 150 kg/hm^2^, N3: 270 kg/hm^2^; P means applying different phosphate fertilizers, P0: 0 kg/hm^2^, P1: 15 kg/hm^2^, P2: 30 kg/hm^2^, P3: 60 kg/hm^2^. Different lowercase letters represent significant differences at the *p* < 0.05 level.

Glutamine synthetase activity (GS) is one of the key enzymes in plants according to assimilation and catalyzes the formation of glutamine from glutamate in plants in the presence of ATP and Mg^2+^. The GS activity of both LAC and HAC types of varieties at different phosphorus levels mostly each decreased gradually with increasing N application. Reaching a maximum under N2P3 treatment, and would have reduced GS activity under high N treatment, which was unfavorable for nitrogen uptake and translocation (Figure 2A). Nitrate reductase (NR) is a key enzyme in plant nitrogen assimilation. The NR activity of both LAC and HAC type varieties increased gradually with increasing N application at different phosphorus levels in most of each. But HAC was inhibited by P2 treatment, and LAC was inhibited by P1 and P3 treatments of high N (Figure 2B).

### 2.6. Effect of Nitrogen and Phosphorus Fertilizers on the Relative Expression of Nitrogen Metabolism Genes in Rice

**Figure 3:**
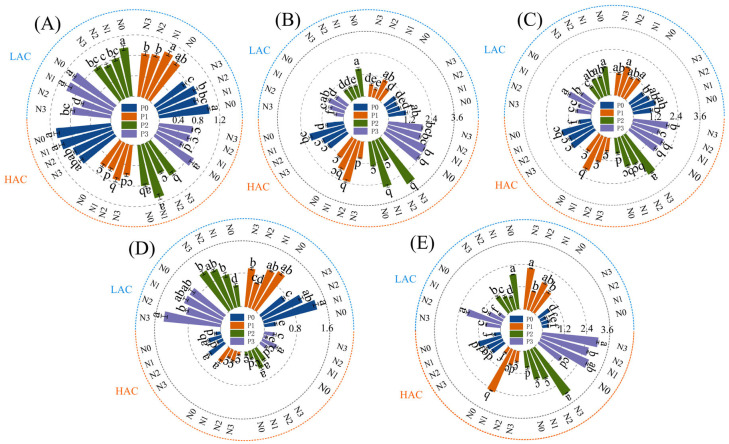
Effect of nitrogen and phosphorus fertilizers on the relative expression of nitrogen metabolism genes in rice. (**A**) Relative expression levels of LAC and HAC *OsGS1;1*; (**B**) Relative expression levels of LAC and HAC *OsGS1;2*; (**C**) Relative expression levels of LAC and HAC *OsGS2*; (**D**) Relative expression levels of LAC and HAC *OsNPF2;2*; (**E**) Relative expression levels of LAC and HAC *OsAMT2;1*. ±: the standard deviations for the mean values. LAC: variety with low amylose content; HAC: variety with high amylose content; N means applying different nitrogen fertilizers, N0: 0 kg/hm^2^, N1: 90 kg/hm^2^, N2: 150 kg/hm^2^, N3: 270 kg/hm^2^; P means applying different phosphate fertilizers, P0: 0 kg/hm^2^, P1: 15 kg/hm^2^, P2: 30 kg/hm^2^, P3: 60 kg/hm^2^. Different lowercase letters represent significant differences at the *p* < 0.05 level.

Effects of nitrogen and phosphorus fertilizers on gene expression of ammonia assimilation-related enzymes, gene expression of ammonium nitrogen transporter proteins, and gene expression of nitrate transporter proteins in rice leaves (Figure 3). *OsGS1;1* and *OsGS1;2* gene expression of LAC and HAC showed differential changes in the two types of varieties at the P2 level. With LAC showing a trend of decreasing and then increasing with increasing nitrogen application, and HAC showing a trend of increasing and then decreasing in increasing with increasing nitrogen application. LAC and HAC showed the same trend in *OsGS2* and *OsNPF2;2* gene expression under high nitrogen and high phosphorus conditions. *OsAMT2;1* gene expression was significantly higher in the P3 treatment, and LAC and HAC in the N2 and N3 treatments, respectively.

### 2.7. Correlation Analysis Between Rice Yield Components and Dry Matter Accumulation Transit

**Figure 4:**
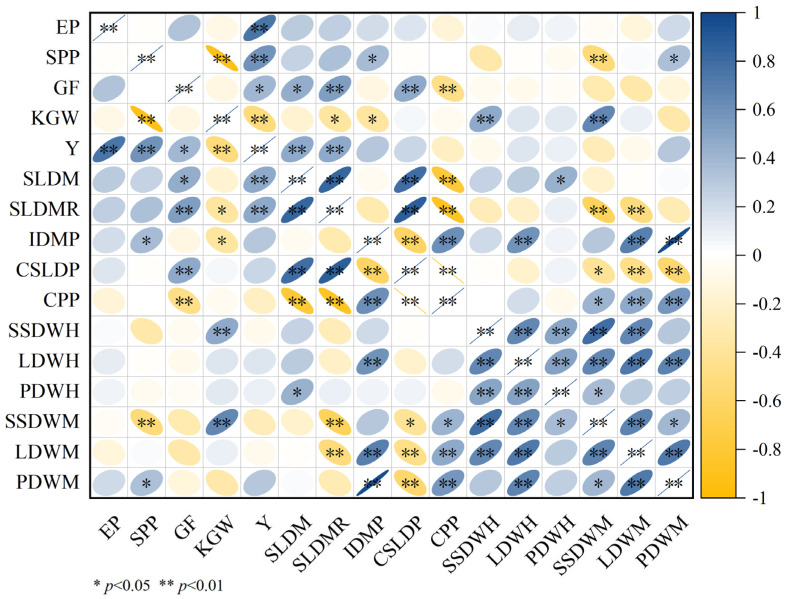
Correlation analysis of rice yield components with dry matter accumulation transit. EP: effective panicle, SPP: spikelets per panicle, GF: grain filling, KGW: 1000-grain weight, Y: yield, SLDM: stem and leaf dry matter translocation, SLDMR: stem and leaf dry matter translocation rate, IDMP: increase in dry matter of panicle, CSLDP: contribution rate of stem and leaf dry matter translocation to the panicle, CPP: contribution rate of photosynthesis to the panicle, SSDWH: stem-sheath dry matter accumulation at full heading stage, LDWH: leaf dry matter accumulation at full heading stage, PDWH: panicle dry matter accumulation at full heading stage, SSDWM: stem-sheath dry matter accumulation maturity stage, LDWM: leaf dry matter accumulation at maturity stage, PDWM: panicle dry matter accumulation at maturity stage. * and ** represent the significance at *p* < 0.05 and *p* < 0.01, respectively.

Significance of nitrogen and phosphorus fertilizers on yield of both types of rice and its yield components (Figure 4). LAC and HAC yields were significantly or highly significantly positively correlated with effective panicles (EP), spikelets per panicle (SPP) and grain filling (GF), Very significant negative correlation with 1000-grain weight (KGW); spikelets per panicle (SPP) were significantly and highly significantly positively correlated with increase in dry matter of panicle (IDMP) and panicle dry matter accumulation at maturity stage (PDWM). It was highly significantly negatively correlated with stem-sheath dry matter accumulation maturity stage (SSDWM); grain filling (GF) was significantly or highly significantly positively correlated with stem and leaf dry matter translocation (SLDM), stem and leaf dry matter translocation rate (SLDMR) and contribution rate of stem and leaf dry matter translocation to the panicle (IDMP); 1000-grain weight (KGW) was highly significantly and positively correlated with stem-sheath dry matter accumulation at full heading stage (SSDWH) and maturity (SSDWM), while it was significantly negatively correlated with stem and leaf dry matter transfer rate (SLDMR) and increase in dry matter of panicle (IDMP). Significant or highly significant positive correlations were found between leaf dry matter accumulation at maturity stage (LDWM) and contribution rate of stem and leaf dry matter translocation to the panicle (CSLDP), leaf dry matter accumulation at full heading stage (LDWH), stem-sheath dry matter accumulation at full heading stage (SSDWH), panicle dry matter accumulation at full heading stage (PDWH), stem-sheath dry matter accumulation maturity stage (SSDWM), panicle dry matter accumulation at maturity stage (PDWM).

## 3. Discussion

### 3.1. Effect of Nitrogen and Phosphorus Fertilizers on Dry Matter Accumulation and Nitrogen and Phosphorus Utilization of Indica Rice with Different Straight Chain Starch Contents

Rice dry matter accumulation affects yield directly or indirectly through fertilizer uptake and translocation [10,27]. Nitrogen and phosphorus fertilizer application directly affects dry matter accumulation and distribution in rice [9,28], however, there are differences in the amount of fertilizer required between varieties [22,29]. Proper fertilization allows rice to absorb more nitrogen and phosphorus for the production of dry matter and promotes the transport of nutrients to the kernel, thereby increasing grain filling and 1000-grain weight [19,30]. Rice is more sensitive to phosphorus application at the full heading stage, and phosphorus deficiency leads to a decrease in leaf photosynthetic rate, inhibition of production and transport of photosynthetic products, resulting in a decrease in the assimilate transport efficiency for grain filling [31,32]. Dry matter accumulation at the full heading stage and maturation stage modulates the photosynthetic contribution rate to the panicle, ultimately impacting the 1000-grain weight (Figure 4). The application of nitrogen phosphorus fertilizers in both rice types increases spikelets per panicle and seed-setting rate by promoting increase in dry matter of panicle and dry matter accumulation during the maturation stage, thereby influencing yield. When phosphorus application was 0 kg/hm^2^, the dry matter accumulation at both the full heading stage and maturation stage in LAC under increased nitrogen application treatments was higher than that in HAC. Additional N fertilization effectively promoted dry matter accumulation in LAC, whereas HAC showed no significant improvement in plant dry matter accumulation with increasing N application rates. Total rice biomass exhibited an increasing trend as N application increased. At the maturation stage, the proportion of panicle dry matter relative to total biomass generally increased with higher N application levels. When phosphorus application reached 30 kg/hm^2^, increased nitrogen fertilization in HAC significantly enhanced efficient dry matter accumulation, and the results of this study are consistent with those of Ye et al. [33], Reasonable application of phosphorus fertilizer can increase the effective tillering and photosynthesis of rice, which can effectively promote the aboveground part of dry matter accumulation. When nitrogen application reached 90 kg/ha, phosphorus supplementation further enhanced crop dry matter production, potentially attributable to improved photosynthetic efficiency [34]. Nitrogen application significantly increased stem-sheath dry matter translocation, translocation rate and translocation contribution to LAC and HAC, thus ensuring contribution rate of photosynthesis to the panicle and ultimately significantly increasing nitrogen and phosphorus utilization. High nitrogen and high phosphorus treatments may lead to imbalance of nitrogen and phosphorus ratios, affecting nitrogen translocation to panicle, which in turn inhibited nitrogen partitioning in panicle at the HAC and LAC flushes, this finding is contrary to the findings of He et al. [35].

### 3.2. Effect of Nitrogen and Phosphorus Fertilizers on Yield of Indica Rice with Different Linear Amylose Contents

Rice yield is not determined by a single factor, but is the result of multi-factor interactions, so to improve rice yield, the influence of various factors should be coordinated [15,27]. Environmental factors such as climate change, precipitation patterns and temperature affect crop growth under different fertilization patterns. Precipitation during the whole reproductive period to the full heading stage affects the increase in rice grain filling and 1000-grain weight, which in turn affects yield [21]. Under phosphorus-deficient or low-phosphorus conditions, rice yield increased with elevated nitrogen application rates. However, under high-phosphorus conditions, yield initially increased but subsequently decreased with increasing nitrogen supply. Notably, at high nitrogen levels, high-phosphorus treatments showed no significant yield differences. appropriate phosphorus application promoted the translocation of nitrogen, phosphorus, and potassium to grains during heading stage, thereby enhancing both grain filling and 1000-grain weight [36,37]. When at high nitrogen levels, the contribution of increased effective panicles and spikelets per panicle to yield is smaller than the effect on yield caused by the reduction in grain filling and 1000 grain weight [38,39]. Nitrogen and phosphorus fertilizers on LAC and HAC affect rice yield mainly through influencing increase in dry matter of panicle, contribution rate of stem and leaf dry matter translocation to the panicle, stem and leaf dry matter translocation, stem and leaf dry matter translocation rate, the accumulation of leaf dry matter at full heading stage, and the accumulation of leaf dry matter at the maturity stage, which in turn affect rice yield by influencing effective panicles, spikelets per panicle, the rate of fruit set, and the 1000-grain weight. When the phosphorus application rate is 0 kg/hm^2^, the single application of nitrogen fertilizer mainly improves the yield by increasing the productive tillers and thus effective panicles; LAC and HAC yields increased with increasing N application at 15 kg/hm^2^ and 30 kg/hm^2^ of phosphorus, and theresults of this study are consistent with those of Zhao et al. [40]. and the application of N fertilizers mainly increased Stem and leaf dry matter translocation and stem and leaf dry matter translocation rate, increase in dry matter of panicle and contribution rate of stem and leaf dry matter translocation to the panicle, and effective panicles and grain filling increase the yields. At a phosphorus application rate of 60 kg/hm^2^, rice yields of both types first increased and then decreased with increasing N application. At a phosphorus application rate of 60 kg/hm^2^, rice yields of both types first increased and then decreased with increasing N application.

The process of nitrogen metabolism is one of the most critical pathways of substance metabolism in plants [28,41], is carried out with the participation of various nitrogen metabolism reaction enzymes [42], it is the metabolic process that catalyzes the uptake, translocation, assimilation and in activation of nitrogen in the plant [34]. With increasing nitrogen application rates, the GS activity in rice leaves generally showed a decreasing trend. Moderate nitrogen fertilization can enhance the activity of nitrogen metabolic enzymes in rice leaves, while excessive application leads to reduced enzyme activity. With the increase of nitrogen fertilizer application, rice leaf GS activity showed an overall decreasing trend, moderate amount of nitrogen fertilizer can improve the activity of nitrogen metabolizing enzymes in rice leaves, but excessive application of enzyme activity instead decreased [43]. Leaf GS activity in rice at all fertility stages tended to increase with increasing N application, but excessive N application resulted in a slower increase in GS activity, and either too high or too low N dosage was detrimental to the improvement of NR activity [44]. The increase in GS and NR activities of LAC and HAC slowed down under high nitrogen conditions, and the application of nitrogen fertilizer under low phosphorus conditions could increase the NR activity of rice leaves, probably to promote the reduction and utilization of nitrate, which would be beneficial for better utilization of nitrogen in rice under phosphorus deficiency, and to promote the growth and increase the yield of rice. The slower increase in *OsSG1;1*, *OsGS1;2* and *OsGS2* gene expression in LAC and HAC under high nitrogen conditions may be related to the fact that excess nitrogen will assist phosphorus in exerting some inhibitory effects on them. Under the same phosphorus level, the expression trends of *OsNPF2;2* and *OsAMT2;1* genes in LAC and HAC varieties were largely consistent, which may be indirectly related to phosphorus influencing the transport and uptake efficiency of ammonium and nitrate in rice.

## 4. Materials and Methods

### 4.1. Test Materials and Sites

**Figure 5:**
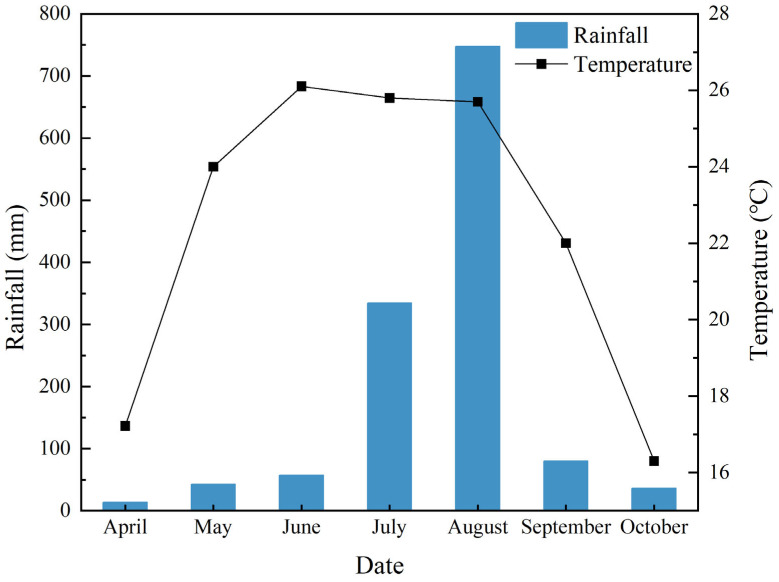
Meteorological data of rainfall and temperature during the 2020 test period.

The experiment was conducted in the experimental base of the Rice Research Institute of Southwest University of Science and Technology, Mianyang City, Sichuan Province, China in 2020.The experiment rice varieties were low amylose rice variety (LAC, Yixiangyou 2115, amylose content is about 16.8%) and high amylose rice variety (HAC, Guangyou 2928, amylose content is about 26.3%). The basic fertility of the test field was 980.23 mg/kg of total nitrogen, 876.53 mg/kg of total phosphorus, 80.2 mg/kg of quick-acting nitrogen, 42.3 mg/kg of quick-acting phosphorus and 76.4 mg/kg of quick-acting potassium. The soil nutrients were determined by the standard procedures [45]. The meteorological data of rainfall and temperature during the 2020 test period are plotted in Figure 5.

### 4.2. Experimental Design

In 2020 two rice varieties were seeded and nursed in trays on April 16 and manually transplanted on 20 May, planting single rice plants in each hole with 0.33 m row spacing and 0.17 m plant spacing. The experiment was conducted in a split-zone design with four different levels of nitrogen and phosphorus (N0: 0, N1: 90, N2: 150, N3: 270 kg/hm^2^) and (P0: 0, P1: 15, P2: 30, P3: 60 kg/hm^2^). The experimental field was divided into 16 areas, each area occupying about 10 m^2^ each, and the areas were poured with concrete so that water and fertilizer would not cascade into each other. Each treatment had 3 replications. Nitrogen fertilizer was applied in the ratio of 7:3, 70% as base fertilizer and 30% as panicle fertilizer; phosphorus fertilizer (calcium superphosphate) and potash (potassium chloride) are used as basal fertilizers before rice seedlings are transplanted, after which the field is treated for uniform water management, pests and weeds according to the actual needs of the field.

### 4.3. Determination of Content and Methods

#### 4.3.1. Rice Yield and Yield Components

At the maturity stage of rice, five representative rice cavities were selected according to the average number of effective panicles, with three replications. The specimens were placed in a dry and ventilated place to dry naturally until the moisture content of the grains was stabilized at 13.5%, and the effective panicles, the pikelets per panicle, the grain filling, the 1000-grain weight and the yield were determined.

#### 4.3.2. Dry Matter Accumulation and Translocation

Three representative plant samples were taken at the flush and maturity stages of rice based on the average effective tillers. The aboveground parts of rice were divided into stems sheaths, leaves and panicles, deactivated at 105 °C for 30 min, dried at 80 °C to a constant weight, and the dry matter weight of each part was measured.

The evaluated by the formulae follows:

Dry matter accumulation at the full heading stage (maturity stage) (kg/hm^2^) = dry mass of stem-sheath at full heading stage (maturity stage) + dry mass of leaves at full heading stage (maturity stage) + dry mass of panicle at full heading stage (maturity stage).

Stem and leaf dry matter translocation (kg/hm^2^) = stem and leaf dry matter accumulation at full heading stage − stem and leaf dry matter accumulation at maturity stage.

Stem and leaf dry matter translocation rate (%) = stem and leaf dry matter translocation/stem and leaf dry matter accumulation at full heading stage × 100%.

Increase in dry matter of panicle (kg/hm^2^) = dry matter accumulation of panicle at maturity − dry matter accumulation of panicle at full heading stage.

Contribution rate of stem and leaf dry matter translocation to the panicle (%) = stem and leaf dry matter translocation/panicle dry matter accumulation from full heading to maturity × 100%.

Contribution rate of photosynthesis to the panicle (%) = 1 − contribution rate of stem and leaf dry matter translocation to the panicle.

#### 4.3.3. Nitrogen and Phosphorus Utilization Rate

At full heading and maturity stages, oven dried samples were ground to pass through a 100-mesh sieve, then digested using the H_2_SO_4_-H_2_O_2_ method for nitrogen and phosphorus content determination in various plant organs, and indicators were determined according to the method of Wolf, B. [46]. Nutrient uptake of each organ was calculated based on the dry matter and nitrogen and phosphorus content of each organ at maturity, in calculating nutrient uptake efficiency and utilization efficiency.

The evaluated by the formulae follows:

Fertilizer harvest index (%) = element accumulation per unit area of plant panicle at maturity/total element accumulation of plant.

Agronomic utilization of fertilizer (kg/kg) = (grain yield in fertilized area − grain yield in blank area)/amount of fertilizer applied.

Fertilizer contribution (%) = (grain yield in fertilized area − grain yield in non-fertilized area)/grain yield in fertilized area × 100%.

Fertilizer uptake utilization (%) = (plant element uptake in fertilized area − plant element uptake in blank area)/fertilizer application × 100%.

#### 4.3.4. Measurement of Enzyme Activity

At the full heading stage, each treatment selected neat and uniformly grown flag leaves to be sampled and put into liquid nitrogen for quick freezing, and then placed in −80 °C ultra-low-temperature refrigerator for preservation. Nitrate reductase (NR) and glutamine synthetase (GS) activities were determined using a microassay method which was determined by the NR and GS kit of Beijing Solarbio Science & Technology Co., Ltd. (Beijing, China), and the unit was Ug^−1^min^−1^ [47,48].

#### 4.3.5. Relative Expression of Rice Nitrogen Metabolism Genes

Flag leaves at the full heading stage of rice were used as test materials, and the samples were put into liquid nitrogen quick-freezing and then quickly transferred to −80 °C ultra-low-temperature refrigerator for backup. The total RNA was extracted from the rice leaves using the RNAprep Pure Plant Kit (Tiangen Biotech Co., Ltd., Beijing, China). cDNA was synthesized using the Evo M-MLV reverse transcription kit (Accurate Biotechnology Co., Ltd., Changsha, China). Gene expression levels were quantified using the SYBR Green Pro Taq HS Premix qPCR kit (Accurate Biotechnology Co., Ltd., Changsha, China) [49] (Table 5).

### 4.4. Data Analysis

The experimental data were collated using Microsoft Excel 2020, analyzed by ANOVA and F-test using SPSS 19.0 statistical software, and multiple comparisons were made at the level of *p* < 0.05 using the least significant difference (LSD) method. Origin 8.5 software was used for graphing.

## 5. Conclusions

Nitrogen fertilizer affects the yield of both types mainly by affecting the effective panicle, the spikelets per panicle and the seeding rate. Phosphorus fertilizer affects the yield of both types of rice mainly by affecting the effective panicl and 1000-grain weight. Under individual phosphorus application level, LAC and HAC had the highest total dry matter and yield at P2 and P1 levels, respectively. Under individual nitrogen application level, LAC and HAC had the highest yield at N3 and N1 levels, respectively. In combination with nitrogen and phosphorus fertilizers, Both LAC and HAC had the highest yield at the N3P1 level. Therefore, to achieve high yields, different fertilization practices should be adopted for different amylose content types in future rice cultivation.

## Figures and Tables

**Table 1:** Effect of nitrogen and phosphorus fertilizers on dry matter translocation in rice.

Treatment	LAC					HAC				
	SLDM/(t/hm^2^)	SLDMR/%	IDMP/ (t/hm^2^)	CSLDP/%	CPP/%	SLDM/(t/hm^2^)	SLDMR/%	IDMP/(t/hm^2^)	CSLDP/%	CPP/%
N0P0	1.67 ± 0.44 f	15.22 ± 3.63 f	7.81 ± 0.86 b	15.02 ± 3.81 e	84.98 ± 3.81 a	2.05 ± 0.72 e	22.49 ± 6.62 ef	6.34 ± 0.38 d	19.21 ± 5.40 e	80.79 ± 5.40 a
N1P0	2.66 ± 0.68 c	23.26 ± 5.95 cd	7.10 ± 0.37 bc	23.9 ± 6.54 cde	76.10 ± 6.57 abc	2.49 ± 0.32 de	26.54 ± 2.45 d	7.41 ± 0.73 cd	23.53 ± 4.85 d	76.47 ± 4.85 b
N2P0	3.14 ± 0.48 ab	25.09 ± 4.96 bc	7.17 ± 0.64 bc	26.57 ± 5.02 bcd	73.43 ± 5.02 bcd	2.60 ± 0.42 de	25.26 ± 2.36 d	8.74 ± 0.73 ab	20.19 ± 3.15 de	79.81 ± 3.15 ab
N3P0	2.73 ± 0.64 bc	21.18 ± 4.56 de	8.72 ± 0.41 ab	20.70 ± 3.92 cde	79.30 ± 3.92 abc	3.58 ± 0.36 ab	32.27 ± 2.47 b	8.39 ± 0.22 abc	29.66 ± 4.61 bc	70.34 ± 4.61 cd
N0P1	2.30 ± 0.58 de	18.58 ± 3.17 ef	7.35 ± 0.43 bc	19.47 ± 4.02 cd	80.53 ± 4.40 ab	2.34 ± 0.08 e	19.90 ± 2.09 f	9.96 ± 0.17 a	16.68 ± 2.66 f	83.32 ± 2.66 a
N1P1	2.79 ± 0.19 bc	25.64 ± 2.78 bc	6.32 ± 0.47 cd	26.32 ± 5.33 bcd	73.68 ± 5.33 bcd	3.09 ± 0.49 bc	34.65 ± 0.98 ab	6.46 ± 0.41 cd	31.69 ± 8.05 ab	68.31 ± 8.05 de
N2P1	3.33 ± 0.76 a	28.08 ± 6.25 ab	5.94 ± 0.48 de	30.98 ± 7.28 abc	69.02 ± 7.28 cde	4.14 ± 0.20 a	35.27 ± 1.39 a	7.04 ± 0.16 bcd	34.84 ± 3.18 a	65.16 ± 3.18 de
N3P1	2.33 ± 0.42 cd	27.48 ± 4.88 b	4.81 ± 0.71 de	30.10 ± 9.16 abc	69.90 ± 9.16 cde	3.33 ± 0.36 bc	29.11 ± 1.12 bc	7.46 ± 0.48 bcd	27.50 ± 1.93 cd	72.50 ± 1.93 bc
N0P2	2.14 ± 0.21 e	17.02 ± 1.24 ef	9.28 ± 0.58 a	16.36 ± 1.00 e	83.64 ± 1.00 a	2.64 ± 0.34 de	23.86 ± 4.03 e	7.40 ± 0.85 bcd	21.58 ± 3.50 de	78.42 ± 3.50 ab
N1P2	2.37 ± 0.29 cd	20.59 ± 3.26 de	6.45 ± 0.14 cd	21.81 ± 4.05 cde	78.19 ± 4.05 abc	3.07 ± 0.29 cd	31.36 ± 3.89 bc	5.96 ± 0.87 d	30.44 ± 4.22 b	69.56 ± 4.22 d
N2P2	3.05 ± 0.31 b	23.69 ± 3.78 cd	7.48 ± 0.79 bc	27.15 ± 4.75 bcd	72.80 ± 4.76 bcd	3.87 ± 0.26 ab	30.32 ± 3.05 bc	8.75 ± 0.18 ab	30.21 ± 4.70 b	69.79 ± 4.70 d
N3P2	3.01 ± 0.28 b	32.28 ± 2.72 a	5.16 ± 0.46 cde	36.26 ± 3.56 a	63.74 ± 3.56 de	3.13 ± 0.22 cd	28.32 ± 2.43 cd	8.74 ± 0.60 ab	23.94 ± 3.47 d	76.06 ± 3.47 b
N0P3	2.70 ± 0.16 bc	23.38 ± 1.69 cd	5.20 ± 0.33 cde	29.97 ± 0.60 abc	70.03 ± 0.60 cde	2.10 ± 0.15 e	23.94 ± 1.08 e	7.21 ± 0.14 bcd	19.93 ± 1.63 e	80.07 ± 1.63 a
N1P3	3.06 ± 0.23 b	28.68 ± 1.99 ab	4.48 ± 0.28 e	34.28 ± 2.65 ab	65.72 ± 2.65 de	2.53 ± 0.41 de	23.63 ± 2.84 e	8.56 ± 0.07 ab	20.68 ± 3.22 de	79.32 ± 3.22 ab
N2P3	3.38 ± 0.40 a	28.60 ± 3.20 ab	6.86 ± 0.31 cd	29.63 ± 4.07 abc	70.37 ± 4.07 cde	3.68 ± 0.15 ab	32.19 ± 2.50 b	7.40 ± 0.34 bcd	31.51 ± 1.52 ab	68.49 ± 1.52 de
N3P3	3.19 ± 0.26 ab	28.62 ± 1.15 ab	6.88 ± 0.72 cd	26.25 ± 1.49 bcd	73.75 ± 1.49 bcd	3.31 ± 0.44 cd	29.91 ± 2.49 bc	7.66 ± 0.56 bcd	28.71 ± 4.67 c	71.29 ± 4.67 bc
N	11.382 **	12.74 **	9.53 **	6.50 **	6.50 **	31.15 **	18.42 **	2.08	13.31 **	13.31 **
P	3.51 *	5.17 **	4.28 **	8.09 **	8.09 **	6.04 **	2.36	0.01	2.68	2.69
N × P	1.11	1.82 *	5.17 **	3.23 **	3.23 **	3.19 **	4.49 **	4.48 **	3.75 **	3.75 **

SLDM: stem and leaf dry matter translocation, SLDMR: stem and leaf dry matter translocation rate, IDMP: increase in dry matter of panicle, CSLDP: contribution rate of stem and leaf dry matter translocation to the panicle, CPP: contribution rate of photosynthesis to the panicle. N, P and N × P denote nitrogen fertiizer, phosphorus fertilizer, and nitrogen and phosphorus fertilizer interactions, respectively. ±: the standard deviations for the mean values. LAC: variety with low amylose content; HAC: variety with high amylose content; N means applying different nitrogen fertilizers, N0: 0 kg/hm^2^, N1: 90 kg/hm^2^, N2: 150 kg/hm^2^, N3: 270 kg/hm^2^; P means applying different phosphate fertilizers, P0: 0 kg/hm^2^, P1: 15 kg/hm^2^, P2: 30 kg/hm^2^, P3: 60 kg/hm^2^. Different lowercase letters represent significant differences at the *p* < 0.05 level. * and ** represent the significance at *p* < 0.05 and *p* < 0.01, respectively.

**Table 2:** Effect of nitrogen and phosphorus fertilizers on nitrogen utilization in rice.

Treatment	LAC				HAC			
	NHI/%	NAE/(kg/kg)	NCR/%	NUE/(kg/kg)	NHI/%	NAE/(kg/kg)	NCR/%	NUE/(kg/kg)
N0P0	64.57 ± 1.21 bcde	--	--	--	72.86 ± 4.74 bc	--	--	--
N1P0	68.77 ± 1.62 ab	9.05 ± 0.02 bc	10.81 ± 0.27 d	11.51 ± 0.19 cd	70.91 ± 2.90 bcd	6.96 ± 0.44 d	6.74 ± 0.18 d	7.32 ± 0.23 d
N2P0	67.02 ± 1.33 bcd	10.83 ± 0.29 b	9.65 ± 0.32 e	13.10 ± 0.03 bc	71.13 ± 3.16 bcd	9.97 ± 0.01 bc	15.1 ± 0.10 bcd	10.8 ± 0.36 bc
N3P0	65.88 ± 2.26 bcde	7.07 ± 0.07 cd	3.25 ± 0.08 f	14.82 ± 0.59 ab	70.47 ± 1.74 bcd	5.57 ± 0.20 d	14.79 ± 0.12 bcd	11.60 ± 0.34 bc
N0P1	52.03 ± 3.67 f	--	--	--	56.58 ± 1.75 f	--	--	--
N1P1	63.12 ± 2.30 cde	2.91 b ± 0.09 f	3.37 ± 0.06 f	7.47 ± 0.22 e	81.15 ± 1.70 a	7.25 ± 0.07 d	11.01 ± 0.01 cd	10.23 ± 0.22 c
N2P1	65.36 ± 2.80 bcde	5.88 ± 0.07 de	10.53 ± 0.16 d	10.28 ± 0.09 d	66.07 ± 2.82 de	13.17 ± 0.16 bc	18.41 ± 0.57 ab	10.04 ± 0.40 c
N3P1	70.94 ± 3.76 a	6.77 ± 0.19 d	21.42 ± 0.16 ab	15.71 ± 0.15 a	74.83 ± 6.82 b	6.08 ± 0.07 d	15.77 ± 0.10 bcd	9.17 ± 0.34 c
N0P2	67.27 ± 1.44 abc	--	--	--	71.73 ± 2.24 bcd	--	--	--
N1P2	64.16 ± 2.81 cde	5.93 ± 0.07 de	6.19 ± 0.06 ab	9.22 ± 0.18 de	69.17 ± 2.16 bcd	9.63 ± 0.15 bc	9.29 ± 0.28 cd	9.67 ± 0.1 c
N2P2	61.69 ± 2.29 e	10.75 ± 0.21 b	16.65 ± 0.21 c	14.69 ± 0.23 ab	60.90 ± 2.16 e	12.64 ± 0.22 bc	18.31 ± 0.5 ab	12.17 ± 0.46 b
N3P2	65.92 ± 2.24 bcde	3.05 ± 0.04 ef	9.27 ± 0.07 e	13.81 ± 0.14 bc	71.18 ± 0.99 bcd	7.56 ± 0.11 d	19.45 ± 0.25 a	16.94 ± 0.04 a
N0P3	62.70 ± 1.74 de	--	--	--	70.58 ± 4.51 bcd	--	--	--
N1P3	64.67 ± 2.10 cde	28.72 ± 0.47 a	28.13 ± 0.16 a	9.25 ± 0.04 de	67.44 ± 2.19 bcd	23.52 ± 0.19 a	20.99 ± 0.18 a	9.45 ± 0.23 c
N2P3	66.48 ± 1.62 bcd	11.00 ± 0.20 b	20.00 ± 0.39 ab	13.24 ± 0.08 bc	66.92 ± 5.28 cd	13.02 ± 0.20 bc	19.62 ± 0.11 a	12.41 ± 0.09 b
N3P3	61.76 ± 1.01 e	5.86 ± 0.12 de	19.3 ± 0.29 bc	14.12 ± 0.14 ab	69.19 ± 2.74 bcd	7.20 ± 0.12 bc	19.53 ± 0.57 a	12.69 ± 0.26 b
N	12.55 **	3.38 *	5.37 **	7.56 **	9.07 **	159.17 **	6.32 **	2.29
P	8.710 **	175.04 **	12.17 **	2.61 *	2.11	1711.46 **	5.80 **	10.08 **
N × P	16.36 **	7.65 **	5.11 **	5.59 **	11.29 **	194.75 **	0.63	2.13

NHI: nitrogen harvest index; NAE: nitrogen agronomic efficiency; NCR: nitrogen contribution rate; NUE: nitrogen uptake efficienc. N, P and N × P denote nitrogen fertilizer, phosphorus fertilizer, and nitrogen and phosphorus fertilizer interactions, respectively. ±: the standard deviations for the mean values. LAC: variety with low amylose content; HAC: variety with high amylose content; N means applying different nitrogen fertilizers, N0: 0 kg/hm^2^, N1: 90 kg/hm^2^, N2: 150 kg/hm^2^, N3: 270 kg/hm^2^; P means applying different phosphate fertilizers, P0: 0 kg/hm^2^, P1: 15 kg/hm^2^, P2: 30 kg/hm^2^, P3: 60 kg/hm^2^. Different lowercase letters represent significant differences at the *p* < 0.05 level. * and ** represent the significance at *p* < 0.05 and *p* < 0.01, respectively.

**Table 3:** Effect of nitrogen and phosphorus fertilizers on phosphorus utilization in rice.

Treatment	LAC				HAC			
	PHI/%	PAE/(kg/kg)	PCR/%	PUE/(kg/kg)	PHI/%	PAE/(kg/kg)	PCR/%	PUE/(kg/kg)
N0P0	62.28 ± 2.32 ab	--	--	--	62.04 ± 2.55 bc	--	--	--
N1P0	57.77 ± 3.19 abcd	--	--	--	72.02 ± 3.99 a	--	--	--
N2P0	55.13 ± 2.01 abcd	--	--	--	62.20 ± 1.53 bc	--	--	--
N3P0	60.43 ± 6.72 abc	--	--	--	70.68 ± 4.54 ab	--	--	--
N0P1	49.47 ± 5.37 cd	11.57 ± 1.1 ab	3.18 ± 0.10 g	3.36 ± 0.08 cd	60.37 ± 0.80 c	7.66 ± 0.32 ab	4.33 ± 0.12 e	2.80 ± 0.02 c
N1P1	61.98 ± 2.81 ab	13.73 ± 0.71 a	4.55 ± 0.02 g	3.97 ± 0.02 bc	61.60 ± 1.23 bc	8.73 ± 0.55 ab	8.22 ± 0.23 c	4.46 ± 0.06 b
N2P1	65.89 ± 0.29 a	14.13 ± 0.40 a	3.22 ± 0.12 g	4.55 ± 0.06 a	62.56 ± 2.25 bc	11.88 ± 0.4 ab	11.74 ± 0.48 b	4.14 ± 0.10 b
N3P1	55.48 ± 5.30 abcd	10.91 ± 0.39 ab	9.93 ± 0.07 b	5.45 ± 0.09 a	60.19 ± 1.43 c	10.04 ± 0.09 ab	2.89 ± 0.04 f	5.51 ± 0.06 a
N0P2	60.81 ± 4.44 abc	13.14 ± 0.42 ab	7.75 ± 0.2 cd	1.63 ± 0.04 gh	61.10 ± 2.03 c	9.42 ± 0.20 ab	6.58 ± 0.10 d	1.87 ± 0.08 de
N1P2	48.76 ± 2.43 d	14.58 ± 0.26 a	12.2 ± 0.22 a	2.42 ± 0.08 efg	60.57 ± 2.31 c	11.59 ± 0.10 ab	3.13 ± 0.15 f	2.44 ± 0.06 cd
N2P2	52.34 ± 2.07 bcd	15.42 ± 0.71 a	13.69 ± 0.25 a	2.79 ± 0.09 de	62.20 ± 4.43 bc	15.45 ± 0.42 a	8.58 ± 0.22 c	2.67 ± 0.05 cd
N3P2	60.60 ± 3.34 abc	11.09 ± 0.43 ab	8.51 ± 0.21 bc	2.64 ± 0.06 def	68.48 ± 2.62 abc	13.12 ± 0.38 ab	4.52 ± 0.08 e	2.7 ± 0.06 cd
N0P3	52.25 ± 6.50 bcd	6.44 ± 0.06 b	5.70 ± 0.15 ef	1.25 ± 0.04 h	67.64 ± 2.33 abc	6.07 ± 0.40 b	11.36 ± 0.36 b	1.17 ± 0.02 e
N1P3	61.09 ± 1.53 ab	9.66 ± 0.54 ab	8.53 ± 0.22 bc	1.50 ± 0.06 h	64.58 ± 4.41 abc	8.44 ± 0.35 ab	9.41 ± 0.07 c	1.52 ± 0.04 e
N2P3	53.65 ± 3.81 bcd	10.08 ± 0.08 ab	6.42 ± 0.15 de	1.96 ± 0.05 fgh	65.00 ± 4.50 abc	8.13 ± 0.49 ab	12.51 ± 0.06 a	1.54 ± 0.03 e
N3P3	54.91 ± 2.44 abcd	8.99 ± 0.02 ab	3.56 ± 0.10 f	1.86 ± 0.01 fgh	59.13 ± 7.11 c	7.38 ± 0.44 b	11.15 ± 0.50 b	1.50 ± 0.02 e
N	0.19	1.57	17.22 **	14.17 **	0.53	1.54	108.33 **	3.66 **
P	1.11	5.83	120.66 **	121.38 **	2.71	3.51 *	279.81 **	11.49 **
N × P	3.31 **	0.23 **	30.47 **	2.06	2.19 *	0.26 *	724.44 **	105.62 **

PHI: phosphorus harvest index; PAE: phosphorus agronomic efficiency; PCR: phosphorus contribution rate; PUE: phosphorus uptake efficienc. N, P and N × P denote nitrogen fertilizer, phosphorus fertilizer, and nitrogen and phosphorus fertilizer interactions, respectively. ±: the standard deviations for the mean values. LAC: variety with low amylose content; HAC: variety with high amylose content; N means applying different nitrogen fertilizers, N0: 0 kg/hm^2^, N1: 90 kg/hm^2^, N2: 150 kg/hm^2^, N3: 270 kg/hm^2^; P means applying different phosphate fertilizers, P0: 0 kg/hm^2^, P1: 15 kg/hm^2^, P2: 30 kg/hm^2^, P3: 60 kg/hm^2^. Different lowercase letters represent significant differences at the *p* < 0.05 level. * and ** represent the significance at *p* < 0.05 and *p* < 0.01, respectively.

**Table 4:** Effect of nitrogen and phosphorus fertilizers on rice yield and yield components.

Treatment	LAC					HAC				
	Effective Panicles /(×10^4^/hm^2^)	Spikelets Per Panicle	Grain Filling /%	1000-Grain Weight /g	Yield /(t/hm^2^)	Effective Panicles /(×10^4^/hm^2^)	Spikelets Per Panicle	Grain Filling /%	1000-Grain Weight/g	Yield /(t/hm^2^)
N0P0	143.96 ±0.01 e	148.17 ±3.67 bc	88.02 ±3.28 bcdef	36.54 ±0.43 bcd	6.86 ±0.32 fg	161.88 ±0.05 c	195.76 ±2.59 de	90.66 ±0.23 abc	30.30 ±0.63 cdef	8.71 ±0.35 ef
N1P0	165.25 ±3.51 d	136.8 ±0.92 f	88.81 ±3.89 bcdef	36.98 ±0.27 bcd	7.42 ±0.20 ef	177.91 ±3.26 b	199.00 ±5.37 cde	93.30 ±1.36 ab	31.14 ±0.91 bc	10.29 ±0.22 bc
N2P0	177.89 ±3.45 c	146.11 ±3.20 bc	92.00 ±0.72 abc	37.25 ±0.70 bcd	8.91 ±0.30 bc	166.25 ±0.58 c	195.78 ±2.31 de	90.76 ±0.54 abc	30.89 ±0.68 bcd	9.13 ±0.03 de
N3P0	197.23 ±1.15 b	152.05 ±5.41 b	90.48 ±2.85 abcd	36.97 ±0.70 bcd	10.03 ±0.19 a	179.90 ±0.02 b	196.83 ±1.07 de	92.28 ±2.98 ab	29.89 ±0.69 ef	9.77 ±0.05 cd
N0P1	161.92 ±0.02 d	135.41 ±1.80 f	88.55 ±1.64 bcdef	36.62 ±0.75 bcd	7.11 ±0.34 f	159.92 ±3.58 c	193.82 ±0.54 e	89.93 ±0.88 bc	30.83 ±0.03 bcde	8.59 ±0.07 f
N1P1	161.92 ±0.02 d	135.56 ±3.97 f	93.25 ±0.90 a	37.03 ±0.42 bcd	7.58 ±0.19 ef	161.90 ±0.03 c	182.74 ±1.29 f	93.70 ±2.14 a	31.26 ±0.33 bc	8.67 ±0.04 ef
N2P1	163.72 ±3.65 d	141.59 ±4.50 cde	89.22 ±1.05 abcde	37.30 ±0.45 bcd	7.71 ±0.26 ef	160.92 ±1.73 c	219.11 ±0.19 a	93.18 ±1.22 ab	31.34 ±0.45 b	10.30 ±0.27 bc
N3P1	203.91 ±0.01 a	134.99 ±2.99 ef	92.22 ±0.57 abc	36.71 ±0.22 bcd	9.32 ±0.18 b	213.89 ±3.46 a	184.73 ±2.94 f	90.75 ±2.24 abc	31.36 ±0.54 b	11.24 ±0.12 a
N0P2	161.91 ±0.01 d	159.47 ±1.82 def	85.63 ±0.47 ef	36.61 ±0.61 bcd	8.09 ±0.26 cd	161.92 ±0.02 c	193.43 ±3.08 e	87.66 ±2.36 bc	30.95 ±0.53 bcd	8.50 ±0.21 fg
N1P2	163.25 ±2.31 d	143.06 ±4.18 cd	88.29 ±3.22 cdef	38.79 ±0.96 a	8.00 ±0.38 de	159.82 ±3.77 c	206.79 ±1.05 b	91.57 ±0.41 ab	30.92 ±0.65 ab	9.36 ±0.02 d
N2P2	181.91 ±1.73 c	146.35 ±6.47 bc	92.56 ±2.12 ab	37.71 ±0.15 b	9.29 ±0.09 b	177.82 ±3.38 b	204.70 ±1.51 bc	92.23 ±2.96 ab	33.02 ±0.79 ba	11.09 ±0.08 ab
N3P2	177.70 ±3.36 c	136.56 ±5.17 bc	91.83 ±0.9 abc	36.38 ±0.29 d	8.11 ±0.36 cd	213.92 ±3.40 a	200.68 ±2.58 cd	87.76 ±1.94 cd	29.61 ±0.16 f	11.16 ±0.35 a
N0P3	125.94 ±0.01 f	170.60 ±2.95 a	84.83 ±2.67 f	36.14 ±0.78 cd	6.59 ±0.25 g	143.92 ±0.02 d	204.057 ±1.13 bc	84.65 ±1.08 d	30.06 ±0.28 def	7.47 ±0.09 g
N1P3	164.58 ±4.61 d	172.39 ±5.55 a	89.23 ±1.11 abcde	36.41 ±0.92 cd	9.22 ±0.25 b	184.24 ±5.85 b	220.68 ±2.07 a	86.01 ±1.02 d	31.05 ±0.23 bcd	10.86 ±0.20 abc
N2P3	177.58 ±3.58 c	143.95 ±3.76 b	89.54 ±1.65 abcde	37.45 ±0.54 bc	8.57 ±0.32 cd	177.87 ±3.54 b	185.36 ±0.31 f	89.73 ±3.33 bc	31.30 ±0.66 bc	9.26 ±0.07 d
N3P3	163.59 ±3.60 d	155.21 ±5.50 ef	87.24 ±2.78 def	36.71 ±0.98 bcd	8.13 ±0.20 cd	161.92 ±0.01 c	209.15 ±1.22 b	91.53 ±0.97 ab	29.45 ±0.25 f	9.13 ±0.19 de
N	477.374 **	6.477 **	4.772 *	8.408 **	66.717 **	131.160 **	0.181	7.471 **	20.944 **	10.207 **
P	87.995 **	2.501	51.708 **	4.421 **	4.478*	15.270 **	0.701	11.831 **	4.857 **	1.737
N × P	78.215 **	2.225 *	12.152 **	2.157 *	21.490 **	42.651 **	1.32	3.610 **	4.153 **	2.322 *

N, P and N × P denote nitrogen fertilizer, phosphorus fertilizer, and nitrogen and phosphorus fertilizer interactions, respectively. ±: the standard deviations for the mean values. LAC: variety with low amylose content; HAC: variety with high amylose content; N means applying different nitrogen fertilizers, N0: 0 kg/hm^2^, N1: 90 kg/hm^2^, N2: 150 kg/hm^2^, N3: 270 kg/hm^2^; P means applying different phosphate fertilizers, P0: 0 kg/hm^2^, P1: 15 kg/hm^2^, P2: 30 kg/hm^2^, P3: 60 kg/hm^2^. Different lowercase letters represent significant differences at the *p* < 0.05 level. * and ** represent the significance at *p* < 0.05 and *p* < 0.01, respectively.

**Table 5:** Primers for qPCR markers for nitrogen metabolism gene expression detection.

Gene Name	Primer
*OsGS1;1*	F: CAAGTCTTTTGGGCGTGATATTGTTGAC
	R: CACCTGATCACCGGCAGAAATGCCGACA
*OsGS1;2*	F: AAAGGCGTTCGGCCGCGACATCGTGGAC
	R: CACTTGGTCAGCAGCGGCGATGCCAACT
*OsGS2*	F: AGAACTTGGACGATGAATCGGGGC
	R: GAGGGAAGGACGCAGGACTGAAGA
*OsAMT2;1*	F: GATGAATCACGCCGAAACAC
	R: GCACGGACGAATCGCTACTT
*OsNPF2;2*	F: GTCGCAGGAGCAAACTAAGCTG
	R: TTTCGCATGTCTCGTTCCCTATG

## Data Availability

The data presented in this study are available on request from the authors.
